# Identification of an Unnatural Sulfated Monosaccharide as a High-Affinity Ligand for Pan-Variant Targeting of SARS-CoV-2 Spike Glycoprotein

**DOI:** 10.1021/acschembio.5c00206

**Published:** 2025-05-13

**Authors:** Ally Thompson, Nehru Viji Sankaranarayanan, John E. Chittum, Virendrasinh Mahida, Sharath S. Vishweshwara, Rakesh Raigawali, Saurabh Anand, Raghavendra Kikkeri, Umesh R. Desai

**Affiliations:** 1 Department of Medicinal Chemistry, School of Pharmacy, 6889 Virginia Commonwealth University, Richmond, Virginia 23298, United States; 2 Center for Drug Discovery, 6889 Virginia Commonwealth University, Richmond, Virginia 23219, United States; 3 Department of Chemistry, Indian Institute of Science Education and Research, Pune 411008, India

## Abstract

Identifying smaller sulfated glycan fragments that recognize target proteins with high affinity is highly challenging. In this work, we show that microarray screening of 53 small glycan fragments helped identify distinct sulfated monosaccharide to tetrasaccharide fragments that bind to multiple isoforms of SARS-CoV-2 spike glycoprotein (SgP) with high affinity. Our library consisted of natural and unnatural glycan sequences with a wide range of sulfation levels. The unnatural features arose from the presence of phosphate or fluoro groups on the natural sulfated GAG scaffold as well as sulfate modification of idose fragments that were monomer to tetramer long. None of the natural glycans yielded much promise, which probably conveys the importance of the polymeric glycosaminoglycan chain in SgP biology. However, the unnatural idose fragments with sulfation at the 2, 3, 4, and 6 positions displayed high affinities (100–500 nM) for wild-type, Delta, and Omicron variants of SgP. The unnatural sulfated idose monosaccharide is the smallest molecule known to date that can be classified as a high-affinity, pan-variant fragment. This fragment is expected to serve as the lead for the design of pan-variant ligands with sub-nM inhibition potency.

## Graphical abstract

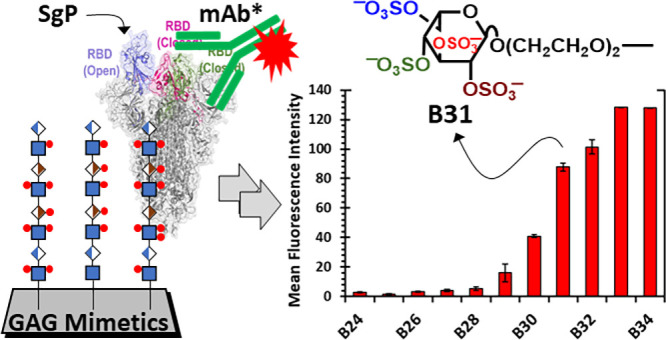

## Introduction

The COVID-19 pandemic, caused by highly transmissible severe acute respiratory syndrome coronavirus 2 (SARS-CoV-2), has caused much devastation in recent times. The virus continues to mutate and infect a large number of people across the globe. Numerous variants, including Alpha, Beta, Gamma, Delta, and Omicron, have now been in circulation, each accompanied by varying types and levels of symptoms and severity.^1,2^ While a number of treatments, including vaccines, antibodies, and small-molecule antivirals, have been put forward, each have been beset with limitations. Vaccines typically focus on disease prevention and may not work effectively for all variants.^3,4^ Likewise, antibodies targeting specific viral proteins may be challenged by the virus’ fast mutation rate. In contrast, small-molecule antivirals typically suffer from off-target toxicity.^5^ *A priori*, an attractive approach is to develop pan-variant inhibitor(s) that may overcome the challenges of virus mutation and unpredictable immune response in many patients. In principle, pan-variant inhibition that works on an early, essential, and obligatory pathway is likely to offer a better approach.

SARS-CoV-2 is a positive-sense RNA virus that is enveloped into a virion and transmitted by respiratory fluid.^7,6^ A distinct feature of SARS-CoV-2 is the transmembrane trimeric spike glycoprotein (SgP) expressed on the virus’ surface that plays an essential role in the host infection.^6–9^ Structurally, each SgP monomer consists of two subunits, S1 and S2. While S1 directly interacts with the host cell surface through its receptor-binding domain (RBD), S2 plays a critical role in membrane fusion.^10^ In the first step, the SARS-CoV-2 virus binds to the angiotensin-converting enzyme (ACE2) receptor located on the host cell surface.^9^ This key step has been found to be dependent upon host cell surface heparan sulfate proteoglycans (HSPGs).^11,12^ Here, the “open form” of the RBD interacts with HSGPs, which further increases its accessibility to ACE2, thereby triggering the internalization of the virus.

HSPGs are transmembrane proteoglycans that possess covalently attached heparan sulfate (HS) chains extending into the extracellular matrix (ECM).^13^ The long polymeric and highly sulfated chains of HS act as recruiting and filtering agents on host cells through which cells, particles, and molecules present in the extracellular milieu are recognized and accepted/rejected. Most enveloped viruses, including SARS-CoV-2, rely on this mechanism to enter into cells. Hence, a simple approach to prevent cellular internalization of enveloped viruses would be to use HS chains as competitive inhibitors, as predicted in the early days of the pandemic.^14^ In fact, nearly two dozen reports since then have concluded that soluble polymeric and oligomeric sulfated agents are effective inhibitors of binding of SgP to ACE2. These include a wide range of natural polysaccharides, or chemically modified derivatives thereof, including heparin/HS,^15–21^ low molecular weight heparins,^17^ chondroitin sulfate E,^22^ pentosan polysulfate,^23,24^ and marine sulfated polysaccharides.^25–28^

Likewise, a range of sulfated oligosaccharides,^15,29–32^ primarily of HS/heparin type, have been studied, of which a sulfated octasaccharide^15^ and a sulfated hexasaccharide^17^ have been proposed as promising ligands of SgP. Although the polymeric chains typically bound SgP (or RBD) with much higher affinity, the sulfated 6- and 8-mers also displayed good binding potencies as high as 50–100 nM,^15,17^ alluding to the high promise of translational outcomes. In a seminal study, Linhardt and co-workers revealed that the SgP of Delta and Omicron variants of SARS-CoV-2 bound heparin much better than the wild-type counterpart.^19^ In a subsequent study, the group concluded that the chain length and sulfation pattern dependence of SgPs of different variants are different.^20^ Interestingly, the minimal chain length for binding to SgP of Delta and Omicron variants was found to be 18-mer, which was significantly different from the prior works showing octa- and hexasaccharides binding potently to wild-type SgP (or RBD).

The concept of inhibiting the first, obligatory step of enveloped virus’ entry into host cells is very attractive. It is also very challenging. The sulfated chains of HS on HSPGs are structurally diverse for good reason. Nature utilizes the sulfate-based electrostatics to recognize many different types of molecules present on cells and particles to ensure rapid recruitment and transmittance of biological signals.^13^ Unfortunately, this introduces a fairly high level of nonselectivity in the first “hand-shake”, i.e., SgP–HSPG system.^33^ This is even more true for smaller sulfated glycans, e.g., glycan fragments, which tend to typically display low affinities.

Our prior work on selectivity principles dictating HS/heparin recognition of different proteins has revealed that within apparently nonselective systems, possibilities exist for identifying unique sulfated structures that exhibit a high level of selectivity for a desired target. We have found this to be the case for heparin cofactor II,^34^ insulin-like growth factor-1 receptor,^35^ and plasmin.^36^ Hence, although challenging, we reasoned that it should be possible to identify sulfated smaller fragments, either natural or unnatural, that recognize SgP with a relatively high selectivity. We also reasoned that these smaller sulfated fragments should be able to engage SgP irrespective of its mutational evolution. If this rationale holds, then it can eventually lead to a synthetic, small HS mimetic that functions as a pan-variant inhibitor. Such an inhibitor would exhibit broad-spectrum action properties under both prophylactic and therapeutic regimes.

In this work, we present microarray-based screening of two groups of synthetic, natural, and unnatural HS mimetics against SgP. We show that molecules as small as sulfated monomers to tetramers bind effectively with various forms of SgP with fairly high potency (<500 nM). This proof of principle, and insights thereof, will help in the design of advanced molecules with much better inhibition potencies.

## Results and Discussion

### Mutational Map of SgP Variants and Possibility of a Pan-Variant Fragment

Because of the role of HSPGs in the early steps of virus internalization, it is crucial to assess how mutational changes in the RBD may impact this recognition. Mapping the electrostatic potential (ESP) is a classic route to predict GAG recognition by proteins.^37^ Hence, the three-dimensional (3D) structures of different RBD variants were modeled and ESP mapped onto the surface (Figure 1). The RBD of the Alpha variant carries a single mutation (N501Y), whereas Beta and Gamma variants have three (E484 K, K417N, and N501Y). In comparison, RBD Delta has two mutations T478 K and L452R, which enhance the surface ESP. In contrast, the Omicron variant has numerous mutations in its RBD including G339D, S371L, S373P, S375F, K417N, N440 K, G446S, S477N, T478 K, E484A, Q493 K, G496S, Q498R, N501Y, and Y505H (Figure 1F),^1,38,39^ which collectively impact the ESP to enhance the potential for engaging negatively charged molecules. It is important to note that although an increase in electropositive character tends to enhance affinity for heparin-like molecules, it does not automatically enhance selectivity of binding. As shown in earlier studies on antithrombin and thrombin,^40^ selectivity relies on multiple features such as multidentate nonelectrostatic forces, reduced gyrational motion, and asymmetric interaction points.

**Figure 1:**
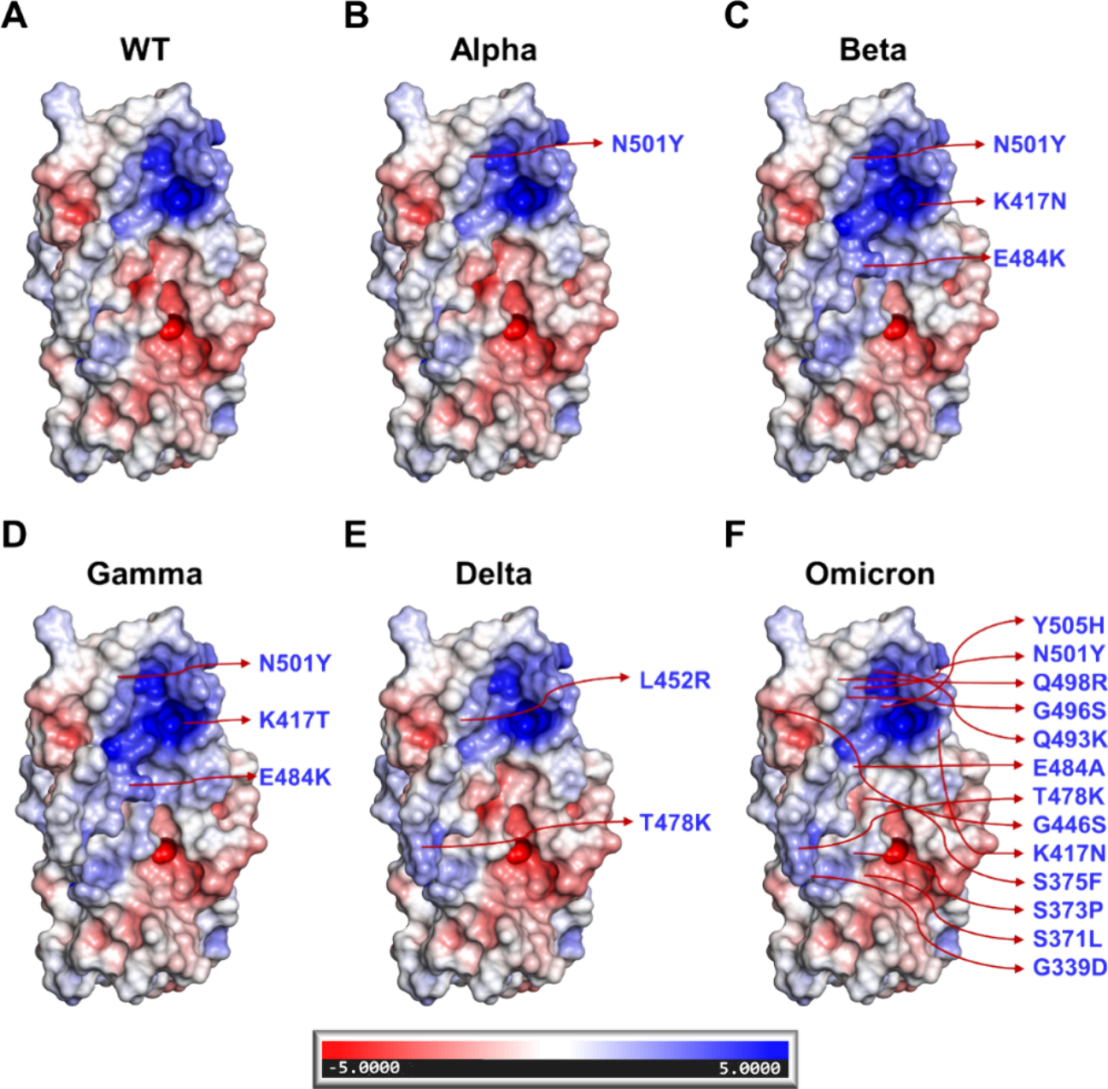
Electrostatic surface potential (ESP) maps of RBD variants (A) wild-type, (B) Alpha, (C) Beta, (D) Gamma, (E) Delta, and (F) Omicron. Also shown are changes in specific amino acid residues in these RBDs. The ESP is mapped in the range −5 to +5, where the red indicates negative charge and blue indicates positive charge distribution.

### Composition of the Library of HS Mimetics **A1**—**A19**

Because we intended to identify smaller fragments with high affinity, we first utilized a library of 19 synthetic mimetics of HS ranging from mono- to tetrasaccharide in length with variation in core sugar scaffold, charge density, and pattern of charges.^41–43^ Two natural GlcNAc/GlcN—IdoA scaffolds (**A1—A4** and **A7—A13**) and one unnatural oligomeric IdoA scaffold (**A14—A19**) were explored (Figure 2). Some of the interesting substitution patterns studied included phosphate groups at the 2- and/or 6-positions (**A5**, **A6**) and 3*-O-*sulfated GlcN residues (**A9**, **A12**, **A13**). The group of unnatural HS mimetics (**A14—A19**) included monomers and trimers of IdoA residues containing variable sulfation at the 2- and/or 4- positions (Figure 2C). Overall, the number of negatively charged groups (sulfate, phosphate, and carboxylate) ranged from a minimum of two to a maximum of seven with variations at the 3- or 6-positions of GlcN and 2- or 4-positions of the IdoA residues. Finally, each synthetic glycan contained an amine linker at the reducing point to allow for covalent attachment to NHS-coated microarray slides.

**Figure 2:**
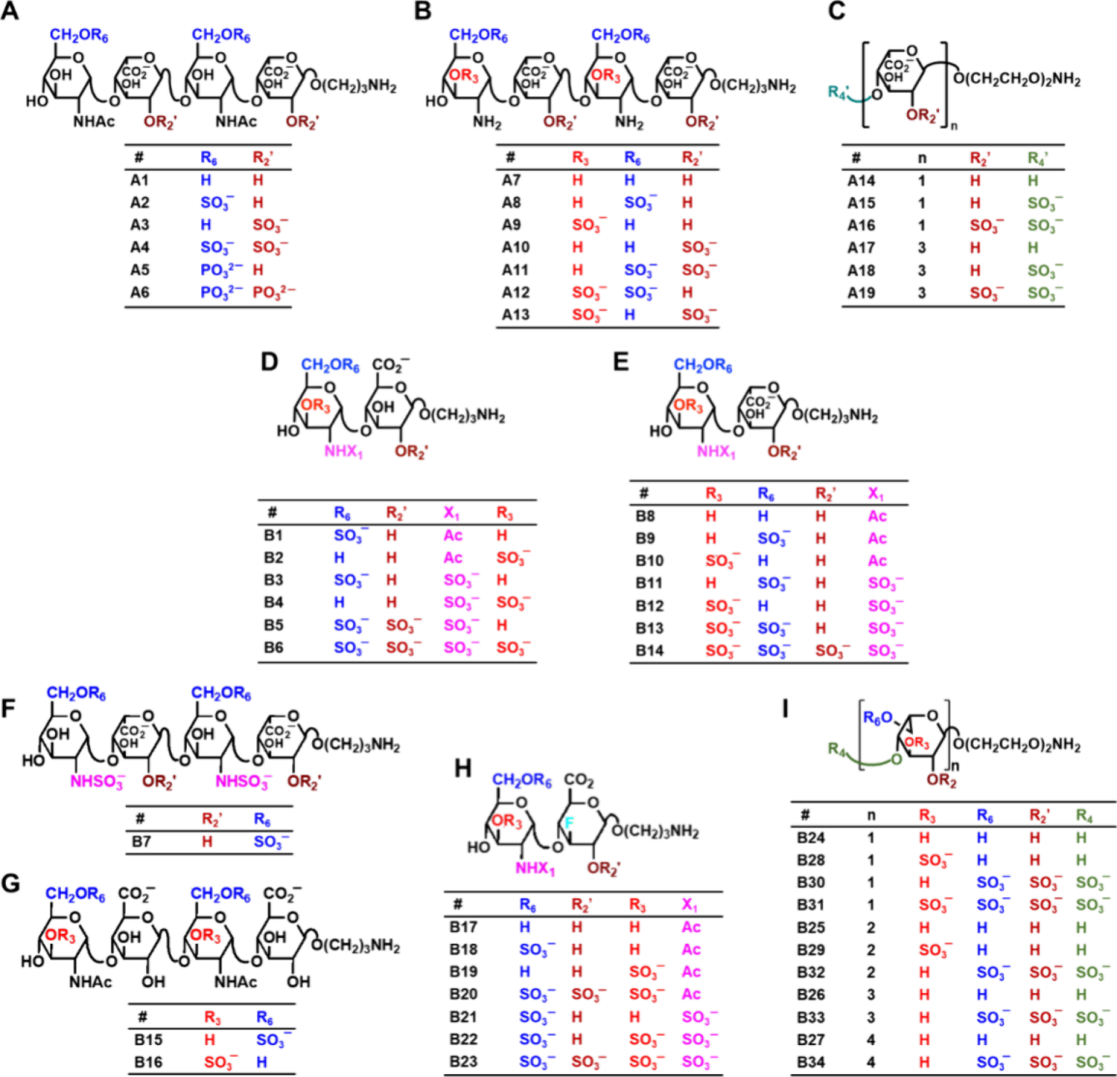
Structures of synthetic HS mimetics A1—A19 and B1—B34. Both libraries of molecules were synthesized earlier using multistep carbohydrate chemistry (see refs 41–43 ). The two libraries consisted of molecules mimicking “natural” glycan sequences (A1—A4, A7—A13, and B1—B16) and “unnatural” sequences (A5—A6, A14—A19, and B17—B34). The natural glycan sequences had either GlcN-GlcA (B1—B6 and B15—B16) or GlcN-IdoA (A1—A4, A7—A19, and B7—B14) scaffolds. The unnatural glycan sequences either had a fluoro group at the 3-position of GlcA (B17—B23) or had an oligo-idose-based scaffold (B24—B34). Each molecule was homogeneous (>95%), characterized using NMR (see refs 41–43 ) and fully water-soluble in buffers used for microarray studies.

### Microarray Screening of Mimetics **A1**—**A19** against SARS-CoV-2 Spike Glycoprotein

HS mimetics **A1** *—* **A19** were immobilized onto NHS-coated glass slides using their reducing end amine and screened against wild-type (WT) His-tagged SARS-CoV-2 SgP RBD, as performed earlier.^29,44^ The His tag on the protein was detected using anti-His antibody carrying the AlexaFluor 488/647 tag. Unfortunately, the majority of mimetics in the library, except for **A9**, presented moderate signal suggesting moderate recognition of the RBD (Figure S1). Within these, there were some distinct binders; e.g., **A16** was ∼3-fold better than its closest neighbors. Yet, inadequate selectivity was apparent despite the diversity of structures. For example, the presence of one or more sulfates (OSO_3_^–^) or phosphates (OPO_3_^2–^) in **A1—A6** made no difference (Figure S1B). The most interesting glycan in this group was **A9**, which was >250% better recognized by WT RBD than the next best molecule (**A10**). Interestingly, **A9** contained 3*-O-*sulfated GlcN, whereas **A10** contained 2*-O-*sulfated IdoA. However, **A13**, which contains both of these groups, exhibited lower MFI in comparison to **A9** (Figure S1A). This alluded to the fact that simplistic additive principles in discovering more potent glycans may not always hold.

### Pan-Variant Screening of Mimetics **A1**—**A19**

The pandemic has shown that the HS-binding property of SARS CoV-2 variants has remained intact and perhaps even improved for Delta and Omicron variants.^19^ Considering that the smaller HS mimetics **A1—A19** present a diverse range of structures, we reasoned that it may be possible to differentiate SgP variants, e.g., WT from the Omicron. Hence, we studied **A1—A19** binding to the RBD, monomeric, and trimeric forms of SgP of the WT, Delta, and Omicron variants.

Figure 3 presents the microarray results. Comparison of the profile of **A1—A19** binding to the WT RBD and WT SgP monomer reveals no major differences, except for **A19**, which appears to bind to the latter protein better (Figure 3A). Likewise, no major binding differences were noted between the WT RBD and WT SgP trimer (Figure 3B). This suggests that each member of the glycan library **A1—A19** prefers to bind in the RBD of SgP.

**Figure 3:**
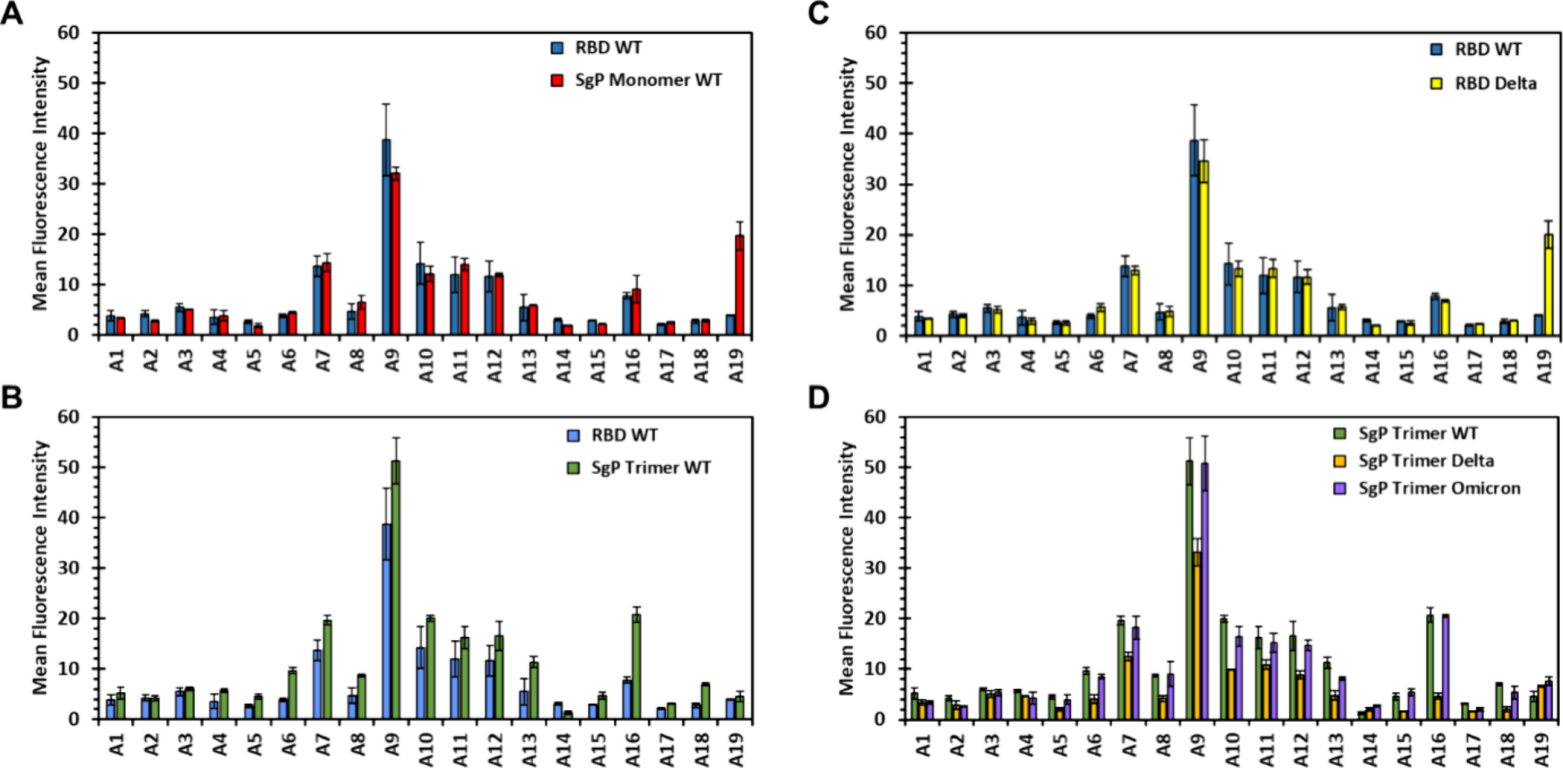
Microarray screening of synthetic HS mimetics **A1**—**A19** against WT, Delta, and Omicron SgP proteins. Comparison of mean fluorescence intensities (MFIs) of the library of molecules **A1**—**A19** binding to RBD and SgP monomer of the WT variant (A), WT RBD and WT SgP trimer (B), WT RBD and RBD Delta variant (C), and SgP trimers of WT, Delta, and Omicron variants (D). Error bars represent ± 1 SEM.

We then compared the binding profile of the WT RBD with the RBD of the Delta form, which yielded no major differences (except for **A19**) (Figure 3C). We then studied the trimeric forms of SgPs of WT, Delta, and Omicron variants. Here, the MFIs of each glycan (**A1—A19**) against WT and Omicron trimers were found to be equivalent, suggesting equivalent recognition (Figure 3D). For the Delta trimer, the MFIs were slightly lower than those WT and Omicron trimers, but that was true for all molecules of the library. This suggested that the anti-His antibody perhaps recognized the Delta SgP trimer slightly weaker than the other two. Thus, counter to our initial expectation, the study showed nearly equivalent recognition of the three variants at the RBD, SgP monomer, and SgP trimer level.

### Computational Study of Binding Site Preference of Mimetics **A1—A19**

Computational studies on sulfated GAGs are challenging, arising from the possibility of promiscuity of binding. Over the past two decades, we have used an in-house technology, called combinatorial virtual library screening (CVLS), to elucidate both affinity and selectivity of HS at the *in silico* level.^45,46^ This technology relies on a genetic algorithm-based search of the conformational space of HS sequences in the putative binding pockets of proteins to help identify preferred binding ligands as well as their corresponding preferred binding sites. We have employed this technology for more than a dozen HS-binding proteins of diverse 3D structures, such as coagulation factors, anabolic enzymes, serpins, tyrosine kinase receptors, growth factors. The algorithm outputs two parameters including GOLDScore (unitless), which is a measure of “*in silico* affinity”, and root-mean-square difference (RMSD in Å^2^) between the bound poses, which is a measure of “*in silico* selectivity”. The ratio of these two parameters (i.e., $\frac{\mathrm{GOLDScore}}{\mathrm{RMSD}}$ ) for each HS mimetic offers insight into the probability of binding with high selectivity.^47^ The higher this ratio, the higher the probability of binding in the putative site of binding. Comparison of these ratios across multiple sites of binding for the same molecule helps to identify the preferred site of binding.

We performed an exhaustive search of the entire SgP trimer surface to locate plausible electropositive pockets that may serve as sites of binding for **A1** *—* **A19**. We identified 22 subsites (Figure S2), each of which contained a group of Arg/Lys/His residues. Among these 22 subsites, we divided the RBD of SgP, which presented a large surface area of 2368 Å^2^ in the “open form”, into three overlapping binding subsites, labeled BS2, BS3, and BS4. Exhaustive docking and scoring studies using the CVLS algorithm yielded preferences for each HS mimetic binding in the 22 putative subsites (Figure S2C). Although some molecules appeared to prefer sites outside the RBD, more molecules preferred **BS2**—**BS4**. For example, the binding site preference, i.e., the $\frac{\mathrm{GOLDScore}}{\mathrm{RMSD}}$ ratio, for **A1**, **A7**, **A14**–**A16**, **A18**, and **A19**, was significantly in favor of BS4. Three other molecules, **A8**, **A10**, and **A13**, preferred BS2 (Figure S2C). Thus, independent computational results supported the microarray results that small HS mimetics **A1** *—* **A19** are likely to preferentially engage the RBD of SgP.

### Search for Better HS Mimetics

To identify molecules that better recognize RBD, we screened an even more diverse library of sulfated glycans. This library contained 34 compounds and consisted of molecules mimicking “natural” HS sequences (**B1**—**B16**, Figure 2D–G) and “unnatural” sequences thereof (**B17**—**B34**, Figure 2H,I). The natural HS sequences had either GlcN-GlcA scaffold (**B1**—**B6** and **B15**—**B16**) or GlcN-IdoA scaffold (**B7**—**B14**). Within these subgroups, the sulfation level varied considerably with 3-, 6-, and N*-*positions of GlcN and/or 2-position of IdoA/GlcA being sulfated. This implies that some of molecules contain residues that are unique and rare in nature, including GlcN3S (**B2**, **B4**, **B6**, **B10**, **B12**—**B14**, and **B16**) and GlcA2S (**B5** and **B6**). It is important to note that both these rare residues have been noted as seats of high selectivity of protein recognition.

The unnatural glycan sequences either had a fluoro group at the 3-position of GlcA (**B17**—**B23**) or had an oligo-idose-based scaffold (**B24**—**B34**). Fluorination of the 3-position of GlcA (Figure 2H) is an unnatural modification but supports hydrogen bonding as well as hydrophobic interaction. In contrast, idose-based **B24**—**B34** oligomers (Figure 2I) are most different from natural HS structures and arguably the most “drug-like” small fragments. One reason for this is the absence of carboxylic acid at the 6-position, which eliminates the conformational flexibility demonstrated by IdoA residues.^48^ Yet, the group of idose oligosaccharides showcases 0 to 9 sulfate groups at 2-, 3-, 4-, and 6-positions, which is the most diverse sulfation level being studied in this work. Overall, the structural diversity embedded in this library of 34 natural and unnatural HS-like sequences offers a higher probability of identifying unique small fragments that bind the RBD of SgP with good affinity and good selectivity.

### Microarray Screening of HS Mimetics **B1**—**B34** against RBD of WT SgP

Glycoarrays were constructed as described above for the first library and used to evaluate the binding of RBD WT (Figure 4). Among the natural disaccharides **B1** *—* **B6** and **B8** *—* **B14**, none stood out as particularly good at recognizing WT RBD. The best molecule was tetrasulfated disaccharide **B14** containing an IdoA residue (Figure 4C). Interestingly, the corresponding GlcA-containing variant **B6** was not as potent (Figure 4B). This suggests the importance of sulfate density rather than the number of sulfates. Among the natural tetrasaccharides (**B7**, **B15**, and **B16**), only **B7** exhibited some preference for RBD WT. Tetrasaccharide **B7** has GlcN-IdoA base scaffold with sulfation at the *N*- and 6-positions. Unfortunately, we did not have access to analog tetrasaccharides with sulfation at *N*-, 2-, 3-, and 6-positions, which precluded detailed structural inference.

**Figure 4:**
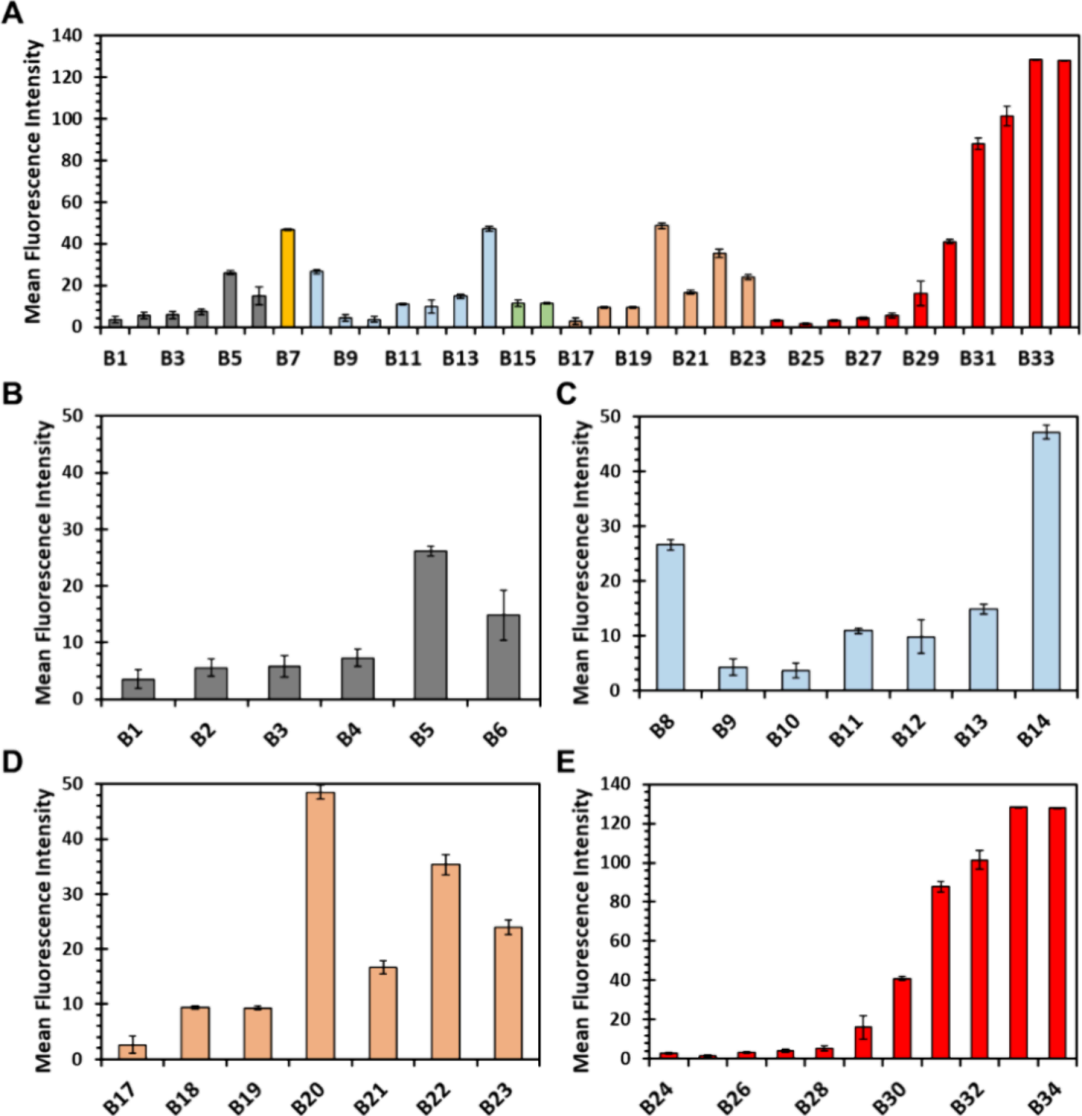
Microarray screening of synthetic HS mimetics **B1**—**B34** against WT RBD of SARS-CoV-2 SgP. Mean fluorescence intensity (MFI) corresponds to the average fluorescence of multiple spots (*n* = 4–6) scaled by the corresponding value of the positive control as the reference, which enables comparison across different library members. Colors segregate the library into different subgroups having the same base scaffold (see Figure 2 for structures). Error bars represent ± 1 SEM.

Among the unnatural glycan fragments containing the 3-fluoro group on the GlcA residue, interesting results were obtained. Disaccharides **B20, B22**, and **B23** exhibited MFIs > 30 (Figure 4D), which were much better than relatively similar disaccharides **B3** *—* **B6** (MFIs < 20) that have a —OH at the same position. It is also important to note that the 3-fluorinated disaccharides are on par in WT RBD recognition as the best tetrasaccharide (**B7**). Not only does this imply the importance the subtle change (—OH → —F), but it appears that this search has identified a novel, small 3-fluoro fragment as a promising ligand of the RBD.

The second group of unnatural idose-based **B24**—**B34** exhibited the most distinctive RBD recognition features (Figure 4E). Of these, **B30**—**B34** were most promising, with MFIs increasing from ∼40 to ∼130 as the chain length increased from monomer to tetramer. Interestingly, each of these molecules contained sulfation at the 6- and 2-positions with terminal sulfation at the 4-position. While **B31** possessed 3*-O-*sulfation, **B32**—**B34** do not. This implied that 3*-O-*sulfate is not a critical group for the idose-based preferred fragment.

A key result from the study on these unnatural glycan fragments is that molecules as small as monosaccharides containing tri- or tetrasulfation, e.g., **B30** and **B31**, can bind RBD with fairly high affinity, whereas trisulfated **B30** displayed MFI 4-fold higher than nonsulfated monosaccharide **B24** and 3*-O-*sulfated **B28** (Figure 4F). Prior to this work, molecules as small as monosaccharides had not been known to bind to the SARS-CoV-2 RBD with reasonable affinity. In fact, a natural sulfated octasaccharide^15^ and a sulfated hexasaccharide^16,29^ have been shown to be minimally necessary. Thus, this work highlights unnatural sulfated glycans as more promising “drug-like” agents for targeting the RBD of SgP.

Trisaccharide **B33** and tetrasaccharide **B34** displayed almost identical MFIs that were higher than those for mono- and di- saccharides **B30**—**B31** (Figure 4F). **B33** and **B34** contain seven and nine sulfate groups, respectively. The similar binding MFIs between the two could indicate that chain lengths greater than trisaccharide and/or sulfation higher than 7 cannot be accommodated into the RBD of SgP. This aspect will need to be studied more with another group of idose-based oligosaccharides containing varying sulfation patterns and density.

### Binding Affinity of HS Mimetics **B31**—**B34** for the RBD of WT SgP

Fragments **B31** *—* **B34** have the potential to bind the RBD with high affinity. To assess that, we first measured their binding affinities using microarrays. The molecules were printed in replicates of four at 100 μM and treated with seven different concentrations of His-tagged RBD. The interaction between the two was quantified by anti-His AlexaFluor 647 antibody. The raw fluorescence images of the microarray are captured in Figure S3, while Figure 5A shows the computed binding profiles for **B31** and **B34**. Most binding profiles were found to be not strictly hyperbolic, as typically observed in solution experiments. We analyzed the profiles using a sigmoidal equation to find *K*_D_s to be 354 ± 25 and 201 ± 64 nM, respectively. Likewise, the affinities of **B32** and **B33** were found to be 243 ± 41 and 307 ± 38, respectively (Figure S4).

**Figure 5:**
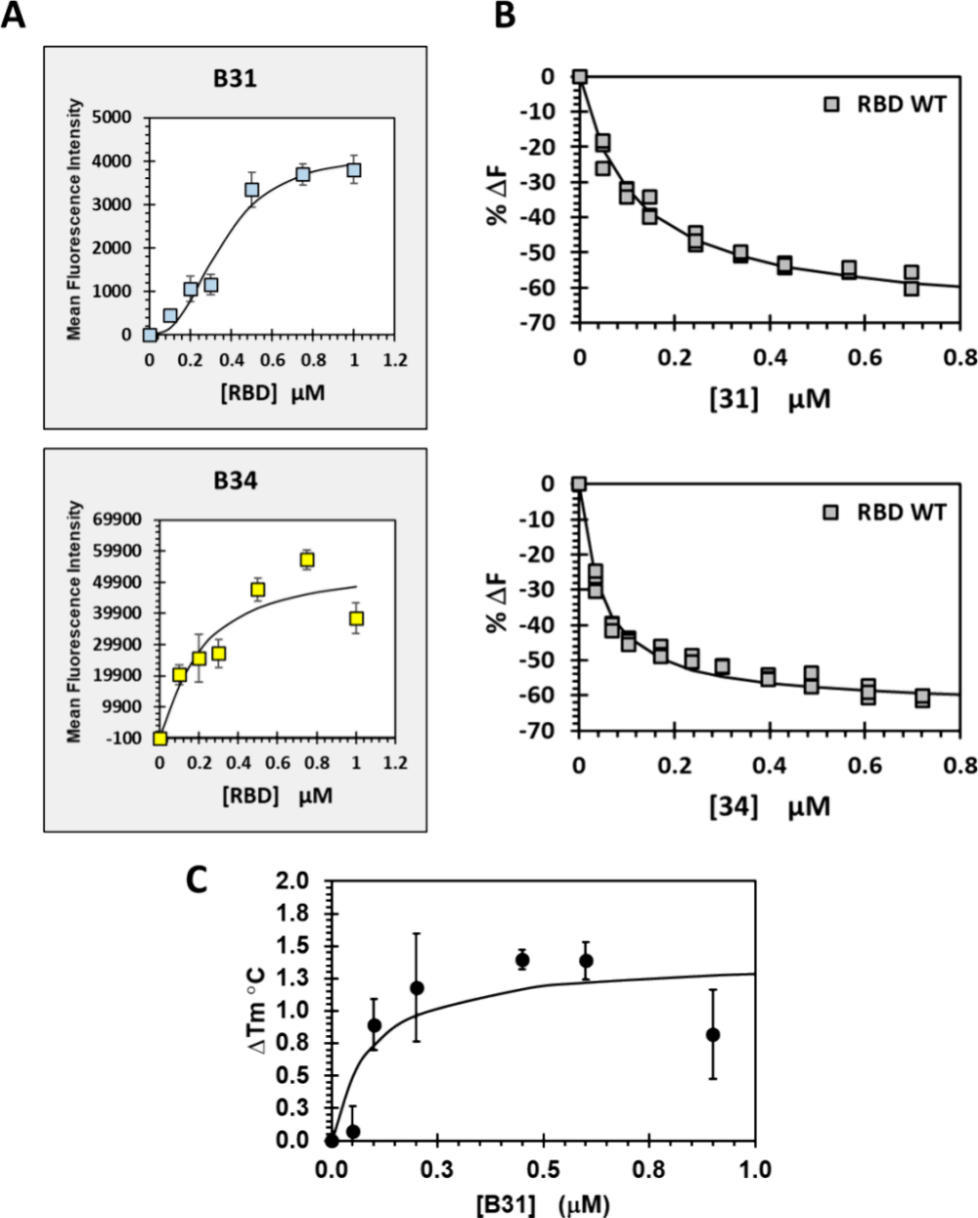
(A) Microarray-based measurement of the binding affinity of small fragments B31 and B34 for WT RBD of SARS-CoV-2 SgP. Mean fluorescence intensities (MFI) were measured at various concentrations of RBD. Error bars represent ±1 SEM. Solid line shows nonlinear fit to the data to obtain apparent *K*_D_. (B) Solution-based measurement of the binding affinity of B31 and B34 for WT RBD of SARS-CoV-2 SgP. Change in fluorescence as a percentage of the initial fluorescence (% Δ*F*) was plotted as a function of the concentration of the titrant. Error bars represent ±1 SEM. Solid line shows nonlinear fit to the data to obtain apparent *K*_D_. (C) nDSF-based titration of B31 and WT RBD of SARS-CoV-2 SgP. The change in the melting temperature as a function of the initial melting temperature (ΔTm) was plotted as a function of the concentration of the titrant. Error bars represent (±1 SE). Solid line shows nonlinear fit to the data to obtain the binding affinity (*K*_D_).

The affinity range (200–350 nM) was very impressive for such small sulfated fragments. To compare, the common HS hexasaccharide was measured to have a WT RBD affinity of 130 ± 58 nM.^29^ This implied that monosaccharide **B31**, which is much smaller than the HS hexasaccharide, is nearly equivalent in affinity. It is important to note that small sulfated glycans generally exhibit weak affinities (>μM) because of fewer interaction points that raise the off-rate of binding. Thus, we wondered whether the immobilized state of mimetics **B31** *—* **B34** was the reason for the high affinity. To rigorously ascertain this, we measured *K*_D_s in solution using fluorescence-based titrations for HS mimetics **B31** and **B34**. Monosaccharide **B31** exhibited a classical hyperbolic change in fluorescence upon binding to WT RBD, which yielded a *K*_D_ of 114 ± 15 (Figure 5B). In contrast, tetrasaccharide **B34** displayed an affinity of 61 ± 8 nM.

Although microarray (Figure 5A) and fluorescence spectroscopy (Figure 5B) are orthogonal biophysical techniques and sufficient for rigorously ascertaining affinities, monosaccharide **B31** presented a highly interesting ligand for WT RBD. Hence, we reasoned that affinity measurement using another orthogonal technique would be beneficial. We performed nano differential scanning fluorimetry (nDSF) titrations, wherein the change in the melting temperature (Tm) of a protein is monitored as a function of ligand concentrations to calculate the affinity of interaction.^49^ Heating WT RBD and **B31** (0.0–0.9 μM) containing solutions from 30 to 65 °C demonstrated a clear increase in Tm (Figure S5), which could be analyzed to yield a binding affinity of 85 nM (Figure 5C). This further confirmed the exceptional binding affinity of monosaccharide **B31** for the WT RBD of SARS-CoV-2 SgP.

### Pan-Variant Potential of HS Mimetics

To assess whether the high WT RBD affinity of HS mimetics **B31** and **B34** carries over to the Delta and Omicron variants, we measured their binding affinities. Monosaccharide **B31** exhibited a classic hyperbolic fluorescence change profile from which its affinities for the Delta and Omicron forms of RBD SgP were calculated to be 135 ± 50 and 413 ± 14 nM, respectively (Figure 6A). Likewise, tetrasaccharide **B34** displayed high affinities of 188 ± 67 and 48 ± 86 nM for the Delta and Omicron variants, respectively (Figure 6B). Comparative analysis shows that fragments **B31** and **B34** possessed high affinities in the nM range for WT, Delta, and Omicron variants of the SgP RBD supporting the concept that glycans as small as a monosaccharide can be developed as pan-ligands. Finally, to a large extent, the smaller fragment **B31** presented slightly lower affinity than tetrasaccharide **B34**, which suggest high potential to design better fragments in this size range (Figure S6).

**Figure 6:**
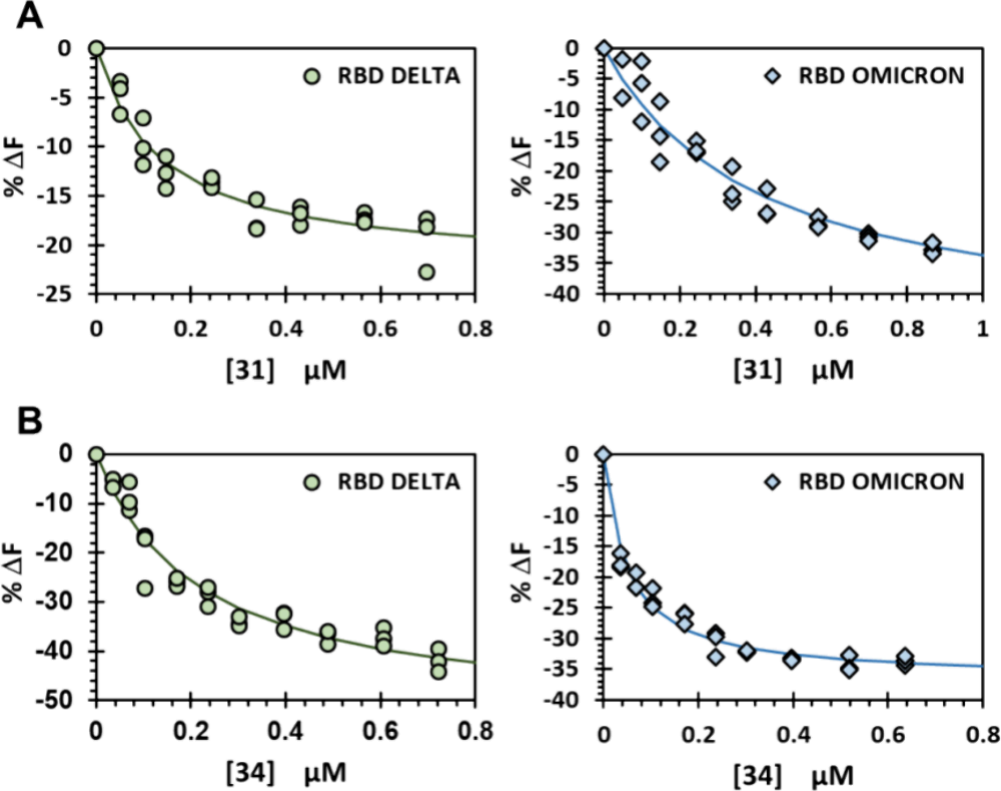
Solution-based spectrofluorometric measurement of the binding affinity of fragments B31 (A) and B34 (B) for Delta and Omicron RBD variants. Change in fluorescence as a percentage of the initial fluorescence (% Δ*F*) was plotted as a function of the concentration of the titrant. Error bars represent ±1 SEM. Solid line shows nonlinear fit to the data to obtain apparent *K*_D_.

Finally, we evaluated the promising fragments **B31** *—* **B34** using a microarray against the Delta and Omicron variants of the SgP trimer as well as RBD to assess their pan-variant potential (Figure 7). The isolated RBD forms displayed increasingly positive trends in binding to the fragments. In the microarray screening, the two variants interacted with **B34** with the strongest intensity. Compared to WT RBD, the Delta and Omicron variants displayed better interaction of roughly 3- and 5-times more for each fragment. Likewise, the trimeric SgP variants also displayed increasingly positive binding trends with HS mimetics in terms of length and sulfation quantity. Interestingly, the trimeric Delta SgP variant displayed a weaker binding potential of each fragment in comparison to the trimeric WT SgP (Figure 7B). Although it is difficult to ascribe an exact reason, perhaps the proportion of the open RBD form in the trimer of the Delta variant is different from the corresponding proportions in WT and Omicron variants.

**Figure 7:**
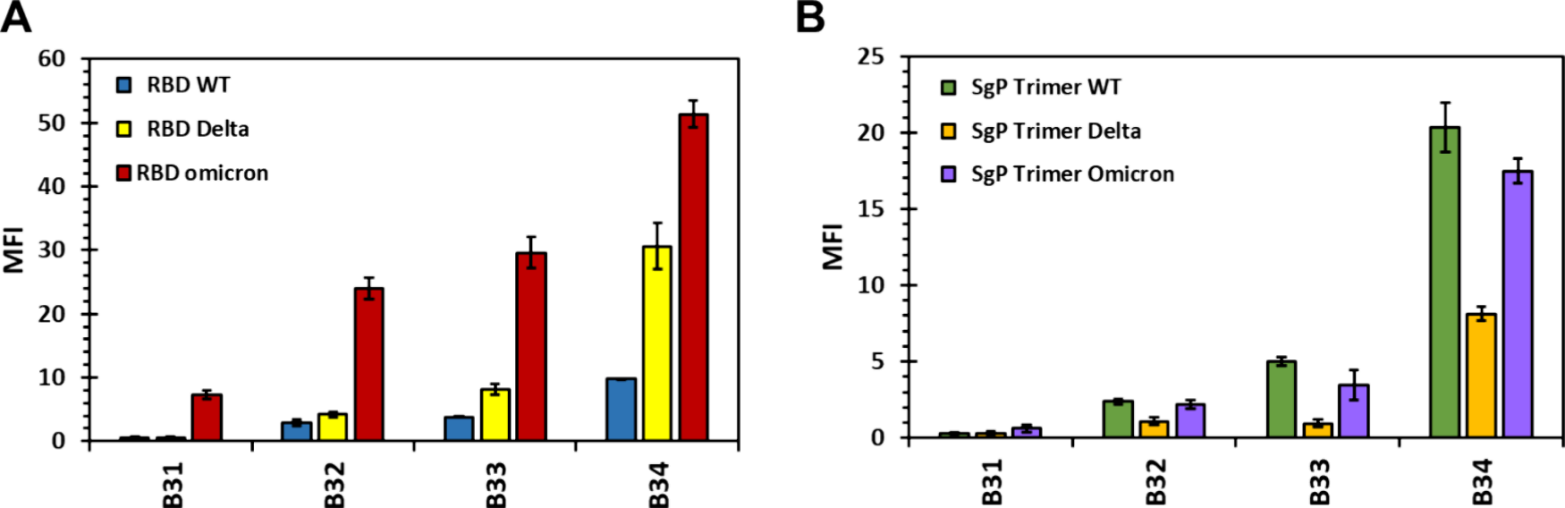
Microarray screening of synthetic HS mimetics B31—B34 against WT, Delta, and Omicron variants of RBD (A) and SgP trimer (B) of SARS-CoV-2. Mean fluorescence intensity (MFI) corresponds to the average fluorescence of multiple spots (*n* = 4–6) scaled by the corresponding value of the positive control as the reference, which enables comparison across different library members. Error bars represent ± 1 SEM.

## Conclusions

Small glycan fragments, such as mono- and di- saccharides, typically do not display high affinity for their protein targets.^50^ Nature utilizes the classic concept of multimerization to enhance the probability of binding as well as selectivity, as evidenced in lectins that recognize monosaccharides such as glucose, galactose, and mannose.^51^ Nature appears to have not developed such a technology for the selective recognition of small GAG fragments, e.g., 2*-O-*sulfated glucuronic acid or 3*-O-*sulfated glucosamine. Instead, nature invokes high-affinity binding, and sometimes selectivity, for target proteins by engineering long chains of sulfated GAGs. Effectively, this implies that sulfated GAG fragments present moderate affinities (>μM) and largely inadequate selectivity. Hence, only a few smaller GAG oligosaccharides have been identified that have high affinities (<μM), e.g., the antithrombin-binding heparin pentasaccharide (50 nM),^52^ IGF-1R-binding hexasaccharide (190 nM),^35^ SgP-binding hexasaccharide (131 nM),^29^ and FGF2-binding tetrasacharide (77 nM).^53^ No examples exist of a sulfated monosaccharide exhibiting high-affinity recognition of its target.

Identifying small high-affinity fragments is challenging but very rewarding. High-quality fragments can form excellent probes for chemical biology. High-quality fragments can theoretically yield promising leads in drug design/discovery. For example, the multimerization of high-quality fragments through conjugation to nanoparticles may lead to therapeutics. Alternatively, if more than one high-quality fragment that engages slightly different binding sites can be identified, it may be possible to use fragment-based drug discovery principles to dramatically enhance affinity and selectivity for the target protein.

This work is a step in the direction of identifying high-quality glycan fragments against different variants of SARS-CoV-2 to develop a pan-variant inhibitor. In this work, we have shown that microarray screening of 53 small glycan fragments led to the identification of a distinct sulfated monosaccharide that binds to the three isoforms of the RBD of SARS-CoV-2 SgP with high affinity. The library of fragments included unnatural as well as natural glycan sequences ranging from mono- to tetrasaccharides containing different levels of sulfation. The unnatural components in this library related to the presence of phosphate or fluoro groups within the natural GAG scaffold. The library also contained idose oligomers of monomer to tetrameric length with variable levels of sulfate groups.

Although interesting recognition features were revealed from the results presented by the natural glycan fragments, none yielded high affinity promise. The unnatural idose fragment with sulfation at the 2, 3, 4, and 6 positions displayed high affinity in the 100–500 nM range for WT, Delta, and Omicron variants of the RBD of SgP. In fact, monosaccharide **B31** is the smallest molecule known to date that can be classified as a high-affinity, pan-variant fragment. We expect this fragment to serve as a foundation for the design of a SARS-CoV-2 pan-variant ligand that exhibits sub-nM inhibition potential.

## Materials and Methods

### Materials

NHS-ester coated slides were obtained from SCHOTT NEXTERION (Rye Brook, New York). Reagents for making the buffers were obtained from Fisher Scientific (Waltham, Massachusetts). Premade buffers for the microarray were from ZBiotech (Aurora, Colorado). Proteins including SARS-CoV-2 SgP RBD (40592-V08B), SgP RBD Delta (40592-V08H90), SgP RBD Omicron (40592-V08H121), SgP monomer (40589-V08H4), SgP trimer (40589-V088B1), SgP trimer Delta (40589-V08H10), and SgP trimer Omicron (40589-V08H26) were obtained from Sino Biological (Wayne, Pennsylvania). Anti-His tag antibodies AlexaFluor 488 and 647 were obtained from BioLegend (San Diego, California). The VERSA10 Spotter microarray printer was from Aurora Biomed (Vancouver, Canada), and the SensoSpot Fluorescence scanner was from Sensovation (Stockach, Germany). The two libraries of carbohydrates used in this study were previously synthesized and described in the works of one the author groups.^41–43^ Each molecule was characterized by using a battery of techniques including ^1^H NMR and HR-MS.

### Microarray Studies

The synthetic carbohydrates were reconstituted in deionized water before aliquoting them in printing buffer (300 mM sodium phosphate, pH 8.4).^29^ The molecules were then spotted in replicates of four or eight on NHS-ester-coated slides using a VERSA10 microarray printer at a relative humidity of 70%. A positive control (PC) consisting of AlexaFluor 488/647-amine and a negative control (NC) consisting of printing buffer alone were also spotted on the slide surface. After printing, the microarrays were kept in the printing chamber at 50% humidity overnight for coupling to occur. The constructed glycoarrays were attached to an eight-subarray slide module fitted with a silicon gasket. To the individual subarrays, 200 μL of glycan array blocking buffer was added. Sealing strips were then applied to the slide modules to prevent cross contamination. The slides were then placed on a shaker (85 rpm) and allowed to incubate with blocking buffer for 1 h, and then the blocking buffer was aspirated in a gentle manner.

His-tagged SgP proteins were reconstituted in ddH_2_O and then aliquoted to 20 μg/mL in the glycan array assay buffer. To each subarray, 200 μL of the protein solution was added. Sealing strips were applied to the slide modules to prevent cross contamination. The arrays were placed on a shaker (100 rpm) to incubate for 1.5 h followed by aspiration of the solution from the subarrays, and 200 μL of the wash buffer (20 mM Tris–HCl, 150 mM NaCl, 0.1% Tween-20, pH 7.6) was added. The arrays were then placed on a shaker (85 rpm) for 5 min, the wash buffer was aspirated, and the wash step was repeated once. Anti-His antibody-AlexaFluor 488/647 was aliquoted in the assay buffer to prepare a stock of 10 μg/mL. To each subarray, 200 μL of the antibody solution was added, sealing strips were applied, and the slides were then wrapped in aluminum foil to reduce photobleaching. Following this, the slides were placed on a shaker (85 rpm) for 1 h. The antibody solutions were then aspirated, the slide modules were removed, and the slides were placed in a Coplin jar that was filled with enough wash buffer to cover each subarray. The jar was wrapped with aluminum foil and placed in a shaker (85 rpm) for 10 min. This wash step was then repeated three times with deionized water and shaking for 5 min. The slides were then dried with a slide spinner centrifuge for 12 s and then stored in a desiccator until scanning.

Scanning of the arrays was done using the SensoSpot reader using “blue” (λ_EM_ = 488 nm) or “red” (λ_EM_ = 647 nm) channels. Following subtraction of background intensity from each raw spot, the intensities were recorded and scaled for standardizing the data across different slides. This scaling factor was calculated from the average intensity of the PC in each subarray, which was set to 100. Experiments were performed at least two times and averaged (±1 SEM).

### Fluorescent-Based Titrations of HS Mimetics B31 and B34 and RBD Isoforms

The fluorescence titrations were performed in phosphate-buffered saline at RT (137 mM sodium chloride, 2.7 mM potassium chloride, 10 mM sodium phosphate dibasic, and 1.8 mM potassium phosphate monobasic, pH 7.4) using a QM4 spectrofluorometer (Photon Technology International, Birmingham, New Jersey). SgP proteins were reconstituted in ddH_2_O, aliquoted to 9.4 μM in phosphate-buffered saline, and administered into a quartz cuvette for a final concentration of 300 nM. Following equilibration (5–10 min), aliquots of HS mimetics (B31 and B34) were introduced into the cuvette followed by gentle mixing and measurement of the emission spectrum in technical triplicate. The spectra were recorded by using intrinsic fluorescent emission (λ_EX_ = 280 nm; λ_EM_ = 340 nm). The excitation slit was set at 0.77 mm, and the emission slit was set at 3.84 mm. Intensities of λ_MAX_ of 340 nm were used in calculations. The change in fluorescence as a percentage of the initial fluorescence (% Δ*F*) was plotted as a function of the concentration of the titrant and analyzed using the standard hyperbolic binding isotherm to calculate the affinity (*K*_D_) using SigmaPlot.

### Nano Differential Scanning Fluorimetry Studies of B31 Binding to WT RBD

The titrations were performed in phosphate-buffered saline (137 mM sodium chloride, 2.7 mM potassium chloride, 10 mM sodium phosphate dibasic, and 1.8 mM potassium phosphate monobasic, pH 7.4) using a NanoTemper Prometheus (NT.Plex) fluorimeter controlled by PR.ThermControl (version 2.3.1). A Discovery scan was first performed to ensure that intrinsic protein fluorescence emission was above 2000 RFU at 330 and 350 nm. WT RBD was introduced into microplate wells so as to reach a final concentration of 0.5 mg mL^–1^ followed by B31 introduction to give 0.0–0.9 μM final concentration. Experiments were done in triplicate. Solutions were heated in Prometheus NT.Plex series nDSF-grade standard capillaries (NanoTemper, Munich, Germany) from 30 to 65 **°**C at a ramp speed of 0.6**°**C/min. Tm values were automatically calculated by PR.ThermControl (version 2.3.1.), and the change in Tm with respect to the Tm of WT RBD alone was plotted as a function of the concentration of the titrant. The results were analyzed using the standard hyperbolic binding isotherm to calculate the affinity (K_D_).

### Preparation of Mimetic Structures for Computational Studies

The carbohydrate HS mimetics (molecules A1–A21 and molecules B22 to B32) were constructed using the BUILD module of SYBYL-X and assigned appropriate atom types. The molecules were then subjected to energy minimization and optimization using the Tripos force field with Gasteiger-Hückel charges, a fixed dielectric constant of 80, and a nonbonded cutoff radius of 8 Å. This process ensured optimal molecular geometries (ring conformations, torsional angles, etc.) for docking studies.

### Preparation of Protein Structure and Definition of Putative Sites of Binding

The three-dimensional structure of the SARS-CoV-2 wild-type spike glycoprotein (SgP) sequence published in the cryo-EM structure (PDB ID: 6VSB) was downloaded for protein preparation.^54^ This structure had several missing residues and loop regions, which were modeled using the SWISS-MODEL web server.^55^ The resulting model exhibited a high sequence identity of 99.2% to SgP and covered 97% of the SgP sequence specifically from amino acids 13 to 1273. The other variants of SgP such as Alpha, Beta, Gamma, Delta, and Omicron were also built using the same method. Subsequently, the modeled structures were processed using Tripos SYBYL-X version 2.2 for further refinement. This preparation involved adding all necessary hydrogen atoms, adjusting the protonation states to ensure correct ionization, assigning appropriate charges to the residues, and performing energy minimization to optimize the structure. During energy minimization with the Tripos force field, the heavy atoms were held fixed, and the minimization was performed for up to 100,000 iterations during which a termination energy gradient of 0.05 kcal/(mol·Å) was used. This ensured stable and well-prepared structures for subsequent molecular docking experiments. To identify potential interaction sites of HS mimetics, the entire trimeric structure of wild-type SgP was divided into 22 distinct putative binding regions as shown in Figure 5A). For each binding region, a radius of 18 Å was used to define an area encompassing the basic residues, which allowed for an unbiased, systematic analysis of the interaction of HS mimetics to SgP.

### Molecular Visualization and Analysis of Different SgP Variants

To understand the molecular features of different SgP variants, the three-dimensional structures of different variants like Alpha, Beta, Gamma, Delta, and Omicron were modeled as detailed above. The 3D models were loaded into PyMOL, and the ESP map of these variants was calculated to highlight the mutations in the receptor-binding domain, as shown in Figure 1.

### Computational Virtual Screening of HS Mimetics against SgPs

The CVLS algorithm, used earlier in multiple studies,^37,46,47^ was employed to elucidate the nature of interaction of the sulfated mimetics with wild-type SgP of SARS-CoV-2. Each molecule was docked into each of the 22 putative binding sites of SgP by using the GOLD v5.6 software. The docking process employed 1000 genetic algorithm (GA) runs, with each run consisting of 100,000 iterations. The GA runs were designed to be exhaustive, wherein early termination was disallowed even if the top three solutions had a root-mean-square deviation (RMSD) of 2.5 Å or lower. At the end of the docking experiment, the two best binding poses were selected for further analysis. Each docking experiment was conducted in triplicate to ensure reproducibility, resulting in at least six binding solutions for each molecule. GOLDScore and RMSD were used to assess the fitness of the docked poses, as described earlier.^37,46,47^

## Supplementary Material



## Notes

Microarray screening of synthetic HS mimetics **A1**—**A19**, 3D visualization of SARS-CoV-2 spike glycoprotein, raw microarray images of **B31**—**B34** binding to WT spike glycoprotein, microarray-based measurement of the binding affinities of **B31**—**B34**, and comparison of binding affinities of **B31** and **B34** for different isoforms of RBD (PDF)

## Abbreviations

ACE2: angiotensin-converting enzyme 2
CVLS: combinatorial virtual library screening
FPX: fondaparinux
GAG: glycosaminoglycan
GlcA: glucuronic acid
GlcN: glucosamine
Hexa: heparan sulfate hexasaccharide
Hp: heparin
HS: heparan sulfate
HSPG: heparan sulfate proteoglycan
IdoA: iduronic acid
RBD: receptor-binding domain
RMSD: root-mean-square deviation
S-AF488: Alexa Fluor 488-labeled streptavidin
SgP: spike glycoprotein
nDSF: nano differential scanning fluorimetry
