# Supplementary material for: Identification of an Unnatural Sulfated Monosaccharide as a High-Affinity Ligand for Pan-Variant Targeting of SARS-CoV-2 Spike Glycoprotein
Source: ACS Chem Biol. 2025 May 13;20(6):1394–405. doi: 10.1021/acschembio.5c00206 (PMC12186262; doi:10.1021/acschembio.5c00206)
Supplement: Supplementary file 1 [file cb5c00206_si_001.pdf]

SUPPLEMENTARY INFORMATION

for

Identification of an Unnatural Sulfated Monosaccharide as a High Affinity Ligand for Pan-Variant Targeting of SARS-CoV-2 Spike Glycoprotein

Ally Thompson,[1,2$] Nehru Viji Sankaranarayanan,[1,2$] John E. Chittum,[1,2] Virendrasinh Mahida,[3] Sharath S. Vishweshwara,[3] Rakesh Raigawali,[3] Saurabh Anand,[3] Raghavendra Kikkeri,[3] and Umesh R. Desai*[1,2]

[1]Department of Medicinal Chemistry, School of Pharmacy, Virginia Commonwealth University, Richmond, Virginia 23298, USA

[2]Center for Drug Discovery, Virginia Commonwealth University, Richmond, Virginia 23219, USA

[3]Department of Chemistry, Indian Institute of Science Education and Research, Pune 411008, India

*Corresponding Author: urdesai@vcu.edu

[$]Equal contribution co-first authors

Sr. No.	Topic	Pg. No.
1	**Figure S1.** *Microarray screening of synthetic HS mimetics A1—A19 against WT RBD of SARS-CoV-2 SgP.*	S2
2	***Figure S2.*** *3D visualization of trimeric form of SARS-CoV-2 spike glycoprotein (SgP).*	S3
3	***Figure S3.*** *Raw microarray slide images of* ***B31—B34*** *binding to WT RBD of SARS-CoV-2 SgP*	S4
4	***Figure S4.*** *Microarray-based measurement of the binding affinity of* ***B31—B34*** *for WT RBD of SARS-CoV-2 SgP.*	S5
5	***Figure S5.*** *Raw nDSF signal (Fluorescence $F_{350/330}$ ratio) for WT RBD and* ***B31*** *binding study.*	S6
6	***Figure S6.*** *Comparison of affinities of* ***B31*** *&* ***B34*** *for different isoforms of RBD.*	S7

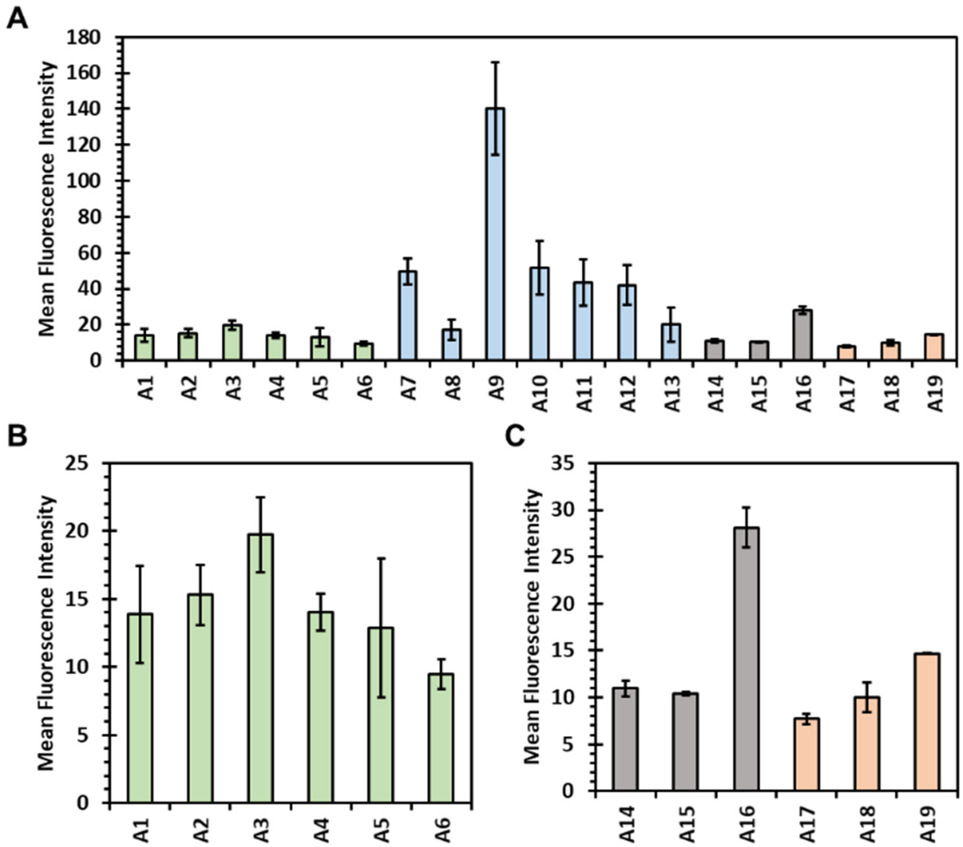

Figure S1. *Microarray screening of synthetic HS mimetics A1—A19 against WT RBD of SARS-CoV-2 SgP. Each mimetic was printed on NHS-glass slides at 100 µM and screened against the target protein using anti-His antibody carrying AlexaFluor 488/647 fluoro-tag. Mean fluorescence intensity (MFI) corresponds to the average fluorescence of multiple spots (n=4–6) scaled by the corresponding value of the positive control as the reference, which enable comparison across different library members. Different colors segregate A1—A19 into sub-groups with identical base scaffold (see* ***Figure 2*** *in the main text for structures). Error bars represent ±1 SEM.*

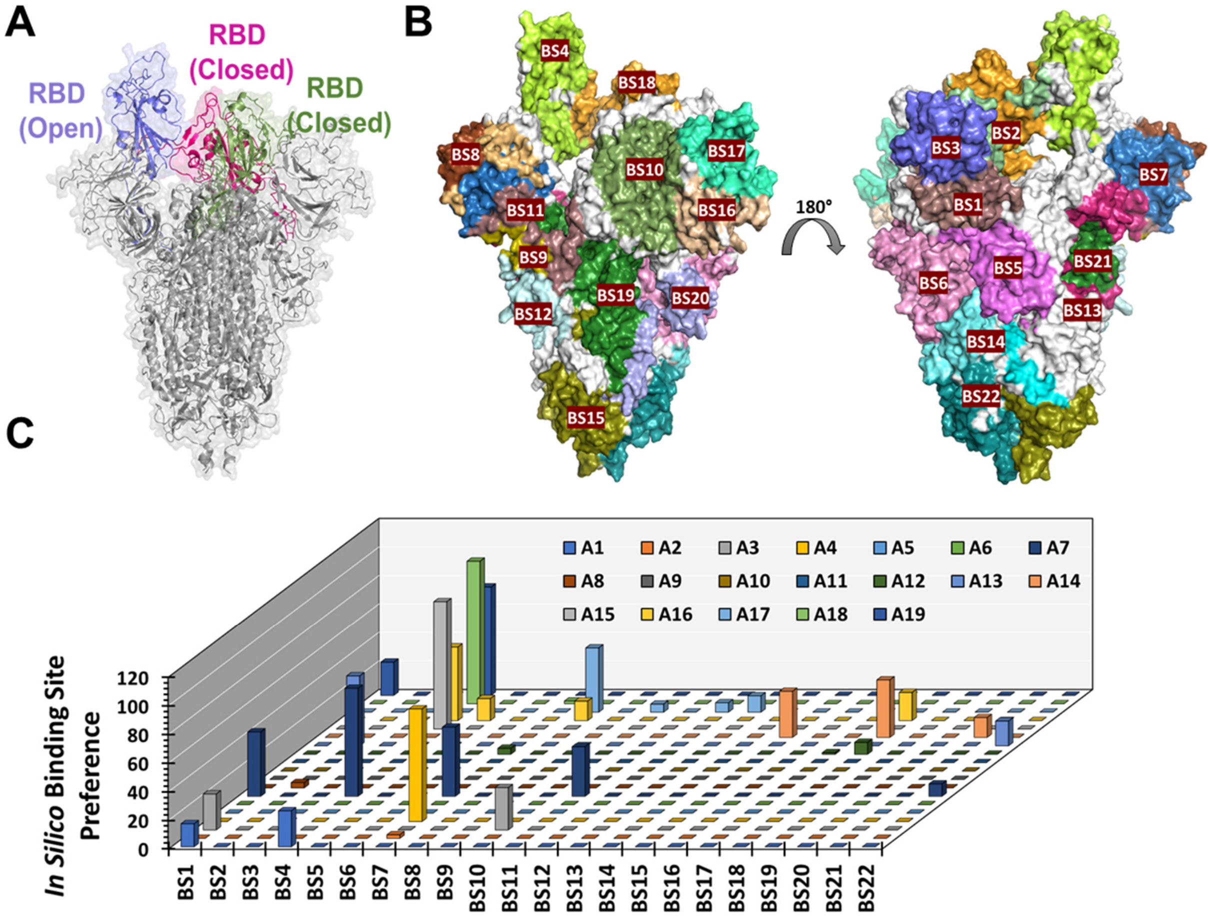

Figure S2. *3D visualization of trimeric form of SARS-CoV-2 spike glycoprotein (SgP). (A) SgP surface, modeled using SWISS-MODELER (PDB ID: 6VSB; cryo-EM structure). The open and closed states of the receptor-binding domain (RBD) in each monomer of the trimeric SgP are illustrated in different colors. (B) The SgP trimer surface divided into 22 binding sub-sites (BS1—BS22) that could theoretically bind sulfated or highly charged species. Both front and back faces are shown. Each binding sub-site BS1→BS22 was defined as a region of ~18 Å radius. The sub-sites are localized in the vicinity of basic residues (Lys, Arg, His) of SgP. (C) Binding site preferences of HS mimetics A1—A19 calculated using an in-house technology called combinatorial virtual library screening (CVLS). This algorithm outputs two parameters including GOLDScore (unitless), which is a measure of "in silico affinity", and root mean square difference (RMSD in Å²) between the bound poses, which is a measure of "in silico selectivity". The ratio of these two parameters (i.e.,* $GOLDScore/RMSD$*) for each HS mimetic offers insight into the probability of binding with high selectivity, i.e., binding site preference (see references 46 & 47 in the main text). See text for details.*

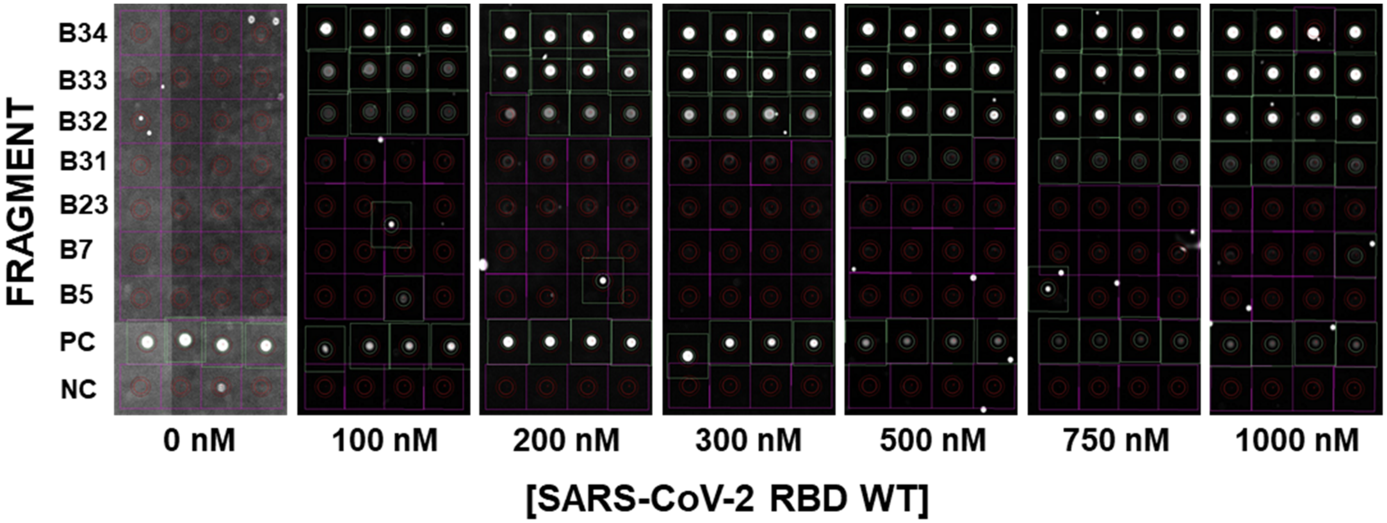

Figure S3. *Raw images of microarray titration of sulfated saccharide fragments binding to WT RBD of SARS-CoV-2 SgP. PC and NC correspond to positive and negative controls, respectively. B5, B7, B23 and B31 – B34 are sulfated fragments (see structures in Figure 2). See Methods for details.*

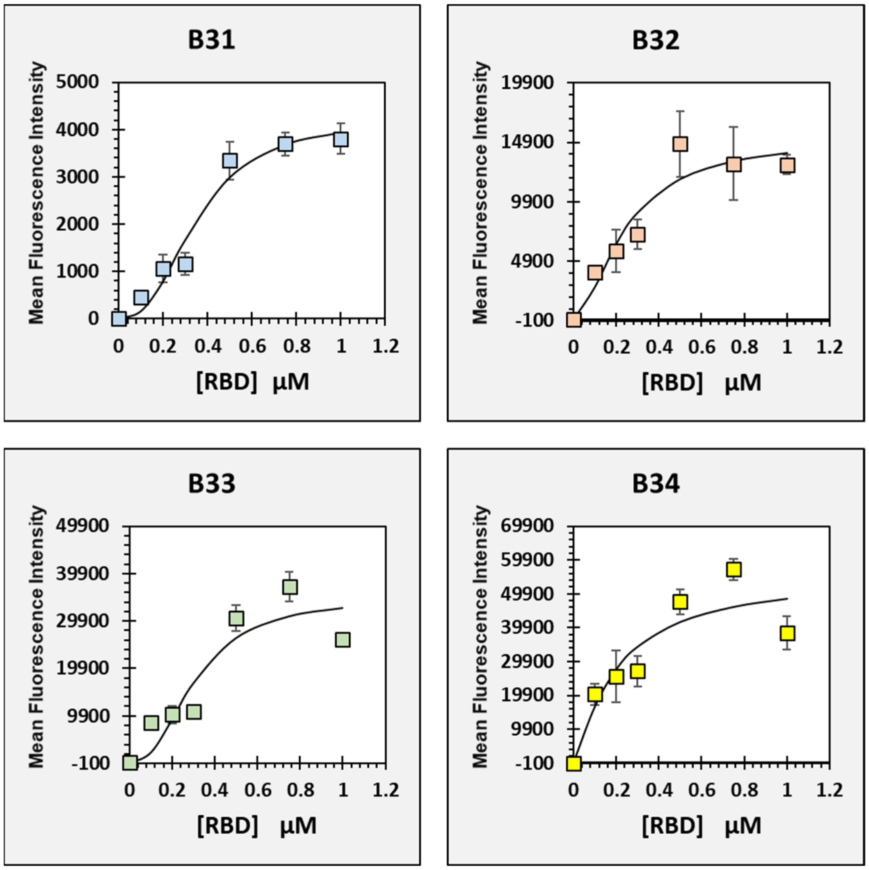

Figure S4. *Microarray-based measurement of the binding affinity of small fragments B31—B34 for WT RBD of SARS-CoV-2 SgP. Mean fluorescence intensities (MFI) were measured at various concentrations of RBD. Error bars represent ±1 SEM. Solid line shows non-linear fit to the data to obtain apparent* K_D*. See Methods for details.*

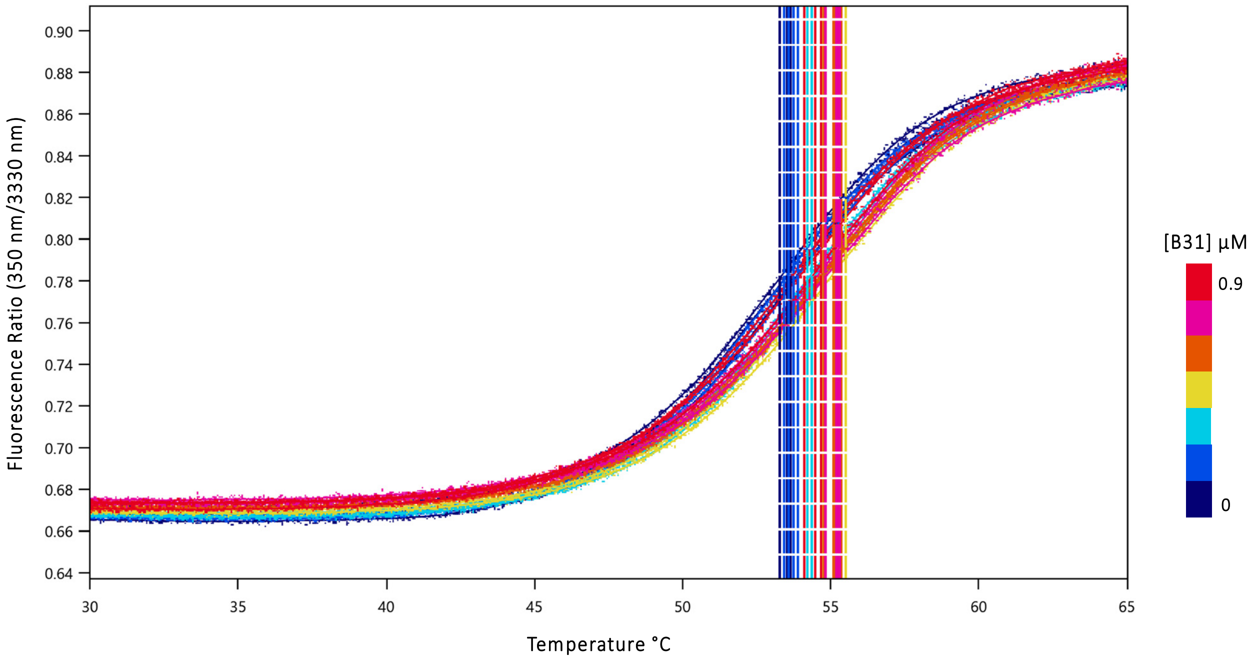

Figure S5. *Raw nDSF signal ($F_{350/330}$ ratio) of 0.5 mg/mL WT RBD of SARS-CoV-2 SgP and monosaccharide* ***B31*** *at 0.0 – 0.9 µM (blue to red) in triplicate from 30° to 65°C at a ramp speed of 0.6°C/min. The ratio of fluorescence at 350 nm to that at 330 nm was utilized to calculate Tm of WT RBD at increasing ligand concentrations, which is visualized by the vertical lines on the graph (same color code).*

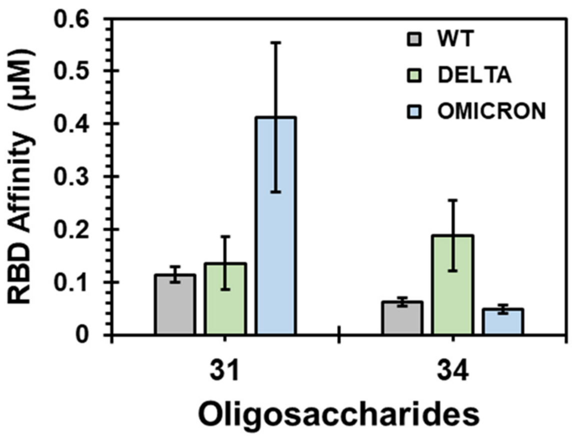

Figure S6. *Comparison of the affinities of B31 and B34 for WT, Delta and Omicron RBD variants.*
